# The major role of toxicology societies in global collaborations – a call to action

**DOI:** 10.1186/2008-2231-20-11

**Published:** 2012-08-28

**Authors:** Jeffrey Brent, Mohammad Abdollahi

**Affiliations:** 1 School of Medicine, University of Colorado, Denver, Colorado, USA; 2 Faculty of Pharmacy and Pharmaceutical Sciences Research Center, Tehran University of Medical Sciences, Tehran, 1417614411, Iran

## 

The science of toxicology in all of its various aspects is, of necessity, a global one. The production and release of potentially toxic chemicals is increasing in many parts of the world. While this can cause local environmental problems, chemical contamination does not respect international and political borders and the resulting health concerns are shared by the global toxicology community. The web-based World Library of Toxicology (http://www.wltox.org), a collaborative effort between Toxipedia.org, the US National Library of Medicine, the International Union of Toxicology, and the Institute of Neurotoxicology and Neurological disorders, was established because of such global concerns. Its intent is to facilitate scientific interchange among various countries. No longer can toxicology function as a fragmented discipline, with individual efforts largely isolated by international borders.

While the concept of the internationalization of toxicology can take the form of collaborative relationships between individual scholars and researchers with overlapping interests, the true potential of such collaborations can best be realized when larger institutions such as governments or professional societies join forces. A classic example of such a collaboration is when the American Academy of Clinical Toxicology (AACT) and the European Association of Poison Control Centres and Clinical Toxicologists (EAPCCT) jointly generated a series of scholarly position statements that resulted in a major upheaval in the approaches to gastrointestinal decontamination in poisoned patients—an upheaval that is still being felt in clinical practice today and one that has enhanced the care of our patients. This collaboration between societies, an important event in the history of clinical toxicology, should serve not just as position statements, but more importantly as an emblematic model of the way professional toxicological societies can interact.

The patterns of international scientific collaboration can be investigated by analyzing scientometric databases for internationally co-authored articles. Such an investigation shows that the science of toxicology is growing globally. For example, when countries from different regions of the world are searched in SCImago for their scientific production in toxicology, then the top countries from 10 regions of the world are as follow: Iran from Middle East, UK from Western Europe, Poland from Eastern Europe, Tunisia from Northern Africa, Nigeria from Central Africa, South Africa from Southern Africa, USA from Northern America, Brazil from Latin America, China from Asiatic Region, and Australia from Pacific region.

If the number of registered toxicologists from societies of these countries from the IUTOX database are counted, an estimated 4000 members are found. The actual number of toxicologists is undoubtedly much higher since many societies are not part of IUTOX. Therefore if only a limited network would be established between those members in these regions of the world, the potential for collaborations on issues such as the care of the poisoned patient, protection of pregnant women, children, and other potentially vulnerable populations from toxic harm in the environment, and better worker protection—all matters of the utmost importance to clinical toxicology—would be great. This is particularly important in medically underserved areas such as many African countries.

We believe that one of the roles of toxicology societies is to follow the example set by the AACT and the EAPCCT to enhance these collaborations. In doing so, different skill sets, expertise and perspectives can be combined to address high-level global challenges. There are many ways societies can do this. The possibilities are so vast that it is beyond the scope of this short call to cooperation to list. Certainly the immediately obvious ones are shared memberships, shared meetings, mutual promotion of activities, joint efforts to fund such activities, the funding and promotion of international research and travel grants, and joint policy statements and practice guidelines about matters of major international importance. Our professional societies can serve as the catalysts and facilitators of these efforts by providing the organizational infrastructure.

The case of the US and Iran provides a salient example of the potential for this kind of collaboration. The challenges of travel between these countries for scientific collaboration are daunting. Despite governmental obstacles, the collaboration between US and Iranian scientists has been steadily increasing. For example, the 2009 10^th^ Iranian Congress of Toxicology, a meeting with excellent scientific content, had several US members on the scientific committee, including one of the authors of this editorial, all unable to attend the meeting because of political issues unrelated to our discipline. The problem of visas for Iranian toxicologists who wish to study, train, and collaborate with colleagues in the US is impeding the natural non-political flow of scientific interaction between colleagues in our two countries. These two societies, however, can go forward where governments cannot. For example, the AACT, an organization with considerable international membership, is advertising the upcoming Irantox meeting on its website (http://www.clintox.org). This gesture, although small in the geo-political quandary of the barriers for collaboration between toxicologists in our two countries, can serve as an example of a first step in a greater relationship between international societies. The same thing exists for Iranian Society of Toxicology (IranTox) that has kept the link of AACT to keep members updated about science of toxicology (www.irantox.ir). There is much to gain from these interchanges. For example, the Iranian model of the care of poisoned patients is oriented around a few specialized treatment centers that see at least of an order of magnitude more patients than the busiest medical toxicology services in the US. This vast wealth of clinical data provides an excellent opportunity for mutual research. The US is one of the goal countries to which talented Iranian scientists and physicians are interested to travel, in the hope that by doing so they may have the potential to achieve better clinical and scientific positions [1].

The internet has provided an invaluable scientific tool for international collaboration by globalizing access to toxicology resources and allowing communication between colleagues irrespective of political boundaries. Most interestingly, as reported by NewScientists [2] and UK’s Royal Society [3], despite political tensions between the USA and Iran, scientific collaboration has proven surprisingly resilient. Between the periods 1996 to 2008, co-authored papers between these two countries increased from just 388 papers to 1,831 [3]. Recent data indicates that Iran’s scientific output rose 18-fold between 1996 and 2008, from only 736 published papers to 13,238 during this time interval, giving Iran has the fastest rate of increase in scientific publication in the world. Among such growth of sciences, the toxicology is listed among the top fields [4-6]. The comparison of drug and poison information centers in Iran and USA is a good example of similarities of scientific concerns between two countries [7]. The enthusiasm for toxicology in Iran, the vast scientific resources and opportunities in the US, and the vibrant cooperation seen between toxicologists in two countries, indicate that this is the time for the AACT and the IranTox move beyond geo-political barriers to establish a more formal collaborative structure. In this respect to increase mutual scientific collaboration between IranTox and AACT, both societies in (January 10, 2012) agreed to share common interests in the academic specialty of toxicology to increase their cooperation in education and research in topics within clinical toxicology wherever it may be deemed possible. They also decided to promote international intellectual exchanges and the scholarly pursuits of members within both organizations in the future.

## Authors’ contributions

Both authors contributed the same, read and approved the final manuscript.

## Authors’ information

Jeffrey Brent and Mohammad Abdollahi are members of American Academy of Clinical Toxicology. Mohammad Abdollahi is president of IranTox.
